# Free Will as a Paradox: Empirical Evaluation of the Construct of Everyday Consciousness

**DOI:** 10.11621/pir2021.0109

**Published:** 2021-06-30

**Authors:** Eugeny L. Dotsenko, Olga V. Pchelina

**Affiliations:** a State University of Tyumen, Tyumen, Russia

**Keywords:** free will, belief in free will, neuroscience, deed (action), decision making, personality, free will as illusion.

## Abstract

**Background:**

Free will belongs to the category of phenomena that are actively discussed in scientific discourse but are neither verified nor proven false. Free will is studied in philosophy, neuroscience, and psychology. We discuss this pluralism, multiplicity of perceptions, and the parties’ arguments in the theoretical part of this article. We approach the existing polemics from the point of view of a person who is in the moment of making a decision and taking responsibility for it. The usual paradoxes are mitigated if we consider free will through the concepts underlying everyday consciousness.

**Objective:**

Our aim is to introduce into the discussion of free will an understanding of its nature as a construct of everyday consciousness, one which acts as a factor in increasing the personal maturity of vital decisions. We also discuss the arguments of the various meta-positions in the dispute about free will.

**Design:**

Our empirical research was designed as a modification of the experiments on imposed attitudes. The sample consisted of 340 people ages 30–50 years.

**Results:**

The level of maturity of actions by the subjects who received the set for determinism was lower than that of the subjects who received the set for free will (U 5133; p = 0.014).

**Conclusion:**

Our study showed that the stronger a person’s belief in free will, the more personally mature that person’s choices — actions — are; and that the more active that belief in free will, the more effective are their efforts to overcome social pressure.

## Introduction

The idea of free will has a long history of discussion which stems from philosophy, but philosophers still do not agree on what free will means. Thus it would be misleading to specify a strict definition of free will since, in the philosophical works devoted to this notion, there is probably no single concept of it (*The Stanford Encyclopedia of Philosophy*).

However, the lack of a common understanding of basic terms is a fairly frequent phenomenon for the sciences. Here the paradox is that not only is the status of the concept a matter for debate, but the very existence of free will as a subject of research remains unclear. On the one hand, this concept, speculative by nature, could remain the prerogative of philosophy: it has no ontological referent, and is not perceived sensorily; it is neither verified nor proven false. On the other hand, the idea is used in many different sciences, probably due to its exceptional social significance.

Free will, defined as “the idea or belief that individual people have volition and the capacity to choose their own courses of action without being fully determined by internal or external forces” (*The Cambridge Dictionary of Psychology*, p. 211–212), is traditionally opposed to rigid determinism, which considers free will nothing more than a subjective illusion. In contrast to determinism, the fundamental nature of the concept of free will is revealed in social practice, both from the side of the actor (whether a person has the right to decide), and from the side of society (whether the person bears responsibility for his/her actions). In this light, the abundance of studies on the attribution of responsibility for offenses is understandable (Nestor, 2018; Anderson, & Kiehl, 2020; Genschow, Rigoni, Hawickhorst, Aschermann, & Brass, 2020; Pundik, 2020), but free will itself has remained outside the scope of research.

The most determined — and most resonant — attempts to deny the existence of free will are made in neuroscience. Many authors (Bargh, 2008; Montague, 2008; Cashmore, 2010; Filevich, Kühn, & Haggard, 2013; Klucharev, 2017) defend the idea of lack of free will. However, even in that camp there is far from unanimity (Bode, Murawski, Soon, Bode, Stahl, & Smith, 2014).

The substitution of a phenomenon caused by terminological coincidence is also very destructive: will-defined as a decision-making process — is mixed up with will — seen as a resource for achieving a set goal. For example, Gailliot, Baumeister, DeWall, Maner, Plant, Tice, Brewer, and Schmeichel (2007) experimentally investigated free will as the source of self-control and concluded that it is a limited resource and, therefore, can deplete. Job, Walton, Bernecker, and Dweck (2013) questioned the depletion effect. As a result, the debate has not been about volition, but about will as an effort to overcome obstacles.

It has already become commonplace to say that “there remains wide-ranging disagreement and confusion” over the concept of free will; that psychologists are exploring “self-control, rational choice, planning, and initiative;” and that “philosophers still debate whether humans truly have free will” (Baumeister, & Monroe, 2014, p. 2). Attempts to substantiate the middle position that person’s decisions are both free and causally conditioned have not yet gone beyond linear ideas.

The unceasing debate over the very existence of free will indicates the need to understand its ontological status as a phenomenon. The **objective** of this current work is to discuss ways to solve this problem: to bring into the discussion an understanding of free will as a construct of everyday consciousness, which acts as a factor in increasing the personal maturity of vital decisions, and to discuss the arguments of the meta-position in the dispute about free will.

### Brain — No Will, No Freedom?

Representatives of the popular and powerful area of neuroscience, which describes mental processes in terms of neural processes, take the extreme position. Based on the natural science tradition, they actually reduce the psyche to brain activity. Nevertheless, we are interested in the trends and the range of arguments they use, which we will briefly outline.

Experiments by B. Libet are often taken as a starting point in reviews. The author himself spoke about the results he obtained with research precision: “Freely voluntary acts are preceded by a specific electrical change in the brain (the “readiness potential” or RP) that begins 550 ms before the act. Human subjects became aware of intention to act 350–400 ms after RP starts, but 200 ms before the motor act” (Libet, 1999, p. 47).

This experimental result was interpreted as evidence of a person’s lack of free will (Tancredi, 2007; Wegner, 2002). V. Ramachandran (2003) in the Reith Lectures said: “All the richness of our mental life — all our feelings, our emotions, our thoughts, our ambitions, our love life, our religious sentiments and even what each of us regards us his own intimate private self — is simply the activity of these little specks of jelly in your head, in your brain.”

The extreme nature of such judgments is easily revealed. Noting the complexity of making arbitrary decisions, L. Deecke identifies three types: What to do, How to do, and When to do.

“After the ‘what to do’ and the ‘how to do’ questions are solved, all that is left to be decided is the ‘when to do’, *i.e.,* to decide the right moment to start the action. This is the task of the frontomesial cortex, including the SMA. The ‘when to do’ is the final question in the motivational chain and is so close to the start of the movement — and time-locked to it — that it can be recorded by the RP^1^ paradigm, while the other two decisions (‘what to do’ and ‘how to do’) cannot be directly investigated by our experimental paradigm” (Deecke, 1996, p. 59-60).

The distinction proposed by Deecke makes it possible to evaluate Libet’s method in a different way: the experimental design prescribed what the subjects should do and how to do it (move a finger), limiting their decision only to a simple “when.” Libet thus reduced the humans’ nature as subjects of volition in two ways, not only by limiting them operationally, but also leaving the motivational and value components outside his research. Yet, these are the components which, in fact, constitute the nature of free will! The experimental hardware creates a scientific aura which masks a serious **methodological error.**

A number of physiologists are quite aware of this. D. Ploog, a researcher on the neurobiological foundations of the behavior of great apes, writes that the problem of will in the aspect of neuroscience is considered “most often with surprising naivete” (Ploog, 2012, p. 439). The author adheres to the principle of causality for the entire spectrum of natural sciences but assures us that the chain of causality “in brain research ends in the immediate past. The principle of cause and effect can only be applied to the past, and not to future events” (*ibid*.). A similar position is taken by the neurophysiologist and primatologist R. Sapolsky, who limits the deterministic influence on the part of neural structures to *one second* (Sapolsky, 2017, p. 26-77).

We should note that there are signs that natural scientists implicitly recognize their basic methodological error. Firstly, interpretations are gradually becoming more cautious and less unambiguous; for example, instead of “neural localization,” the term “neural correlate” is now used (Polák, & Marvan, 2018; Koch, Massimini, Boly, & Tononi, 2016). Secondly, in improving their experiments, supporters of the natural science paradigm have begun to provide subjects with more and more freedom, bringing laboratory conditions closer to natural ones (Perez, Mukamel, Tankus, Rosenblatt, Yeshurun, & Fried, 2015). Thirdly, hoping to get experimental designs that are free from criticism, a number of researchers have turned to simpler processes, sometimes expanding their understanding of volition so much that they go beyond the phenomenon of free will. One such focus is the study of “perceptual decisions” (Bode et al., 2014).

Libet’s idea has also been developed to the point that, although the individual’s decision is allegedly being prepared without the participation of consciousness, once having realized it, the subject can stop its implementation. The subjects of the experiment “were able to exert a veto within the interval of 100 to 200 msec. before the pre-set time to act” (Libet, 1999, p. 51). Here the author of the acclaimed experiments moved from neurophysiology to behavioral phenomena: “All of us, not just experimental subjects, have experienced our vetoing a spontaneous urge to perform some act” (*ibid*.). But “vetoing” means freely realizing one’s will, taking responsibility.

There are some very discouraging results (almost in Deecke’s terms): “The RP is predictive with regards to the whether and the when, if a known task is performed, but not with regards to the what of the action” (Brass, & Haggard, 2008). It doesn’t get any clearer than that. There is an obvious recognition of the limitation on the part of the natural science paradigm, the methodological core of which, within neuroscience, is the model of “computer metaphor.”

Probably the controversy over interpretations comes to a dead end because “we are looking in the wrong place”: it is difficult to find free will in the body, where it most likely cannot exist. This is where the real paradox comes in! After all, none of the adherents of the deterministic paradigm think of looking for the content of a text written in a word processor on the level of distribution of electronic processes in computer boards, or in the structural elements of a matrix. However, it is this paradoxical logic that is practiced in relation to the brain.

### Is the Soul Free?

Distancing ourselves from biological reductionism to consider the problem of the existence of free will, we move on to an alternative logic, a philosophical understanding of the indicated phenomenon. This alternative consists not so much in re- interpreting the facts as in the choice of basic assumptions. R. Smith (2015), a historian of science, is sure that ascribing free will to neural processes is a categorical mistake, since the language of free will is a psychosocial category and an attribute of people; agency is not something attributable to brains or bodies. The study of agency is hence the study of how people attribute freedom, obligation, and responsibility.

But philosophers also cannot ignore Libet’s experiments. Professor A. Mele sees no reason why these experiments should make us doubt the existence of free will. Mele (2006) distinguishes between urges, intentions, and decisions, and divides the latter into distal (related to the future) and proximal (related to the present). This distinction allows him to more accurately analyze experiments: 1) Nothing warrants Libet’s claim that, starting around -550 ms, type II RPs are correlated with decisions or intentions rather than with, for example, urges strong enough; 2) B. Libet investigated only the proximal section of a decision, thus leaving the distal decision to participate in the experiment at all outside the scope of his research. “Brain activity preceding conscious decisions reflects the decision process rather than its outcome. Furthermore, the decision process is configured by conditional intentions that participants form at the beginning of the experiment” (Brass, Furstenberg, & Mele, 2019).

Although the philosopher N. Elzein believes that there are compelling reasons to embrace free will skepticism, “these reasons have little to do with the presence of unconscious precursors to the decisions we make” (Elzein, 2020, p. 16).

One of the most influential libertarians today, R. Kane (1994), defines free will as the ability to be the absolute creator and engine of one’s own goals and intentions. The physical mechanism for the realization of free will as freedom of choice could be quantum effects in the brain. The decision will not be random, if the situation of quantum uncertainty in the brain corresponds to the situation of psychological struggle when the agent is torn between conflicting motives. It is worth emphasizing that Kane continues to search for physical mechanisms of free will, as if there is a direct connection between them and free will.

Thus, we continue to track the dispute between supporters and opponents of the existence of free will, begun by ancient philosophers, the essence of which was formulated in an extremely condensed form by I. Kant in his third antinomy: there is freedom in the world — there is no freedom in the world, but only causality reigns. The very presence of antinomy — the contradiction between two equally provable statements about a subject — testifies, it seems, to the fundamental unverifiability of the statements. Kant “concludes that freedom is based not on knowledge, but on belief. So, he puts belief above knowledge” (Kalinina, 2009, p. 256).

The **paradox** of the problem of free will is that, despite the formulation of the antinomy on the *epistemological* plane, attempts have been made to solve the problem on the plane of *ontology* for 200 years.

S. Harris (2012) argues that “free will is actually more than an illusion (or less).” Hence, either our desires are conditioned by previous experience, and we are not responsible for them, or they are chance-dependent, and we are not responsible for them either. However, this illusion has an active potential (both constructive and destructive). Therefore, to S. Harris’s formula “more than an illusion,” we add a paraphrase: ... we embody our desires into life activity, **turning the illusion into reality.**

Thus, the research question of free will is not solved *with the help of belief*, but it is *thanks to it* that free will is constituted. Free will is generated by the very act of believing in it.

### Free Will is Generated by the Person

We intend to test the lateral (it would be excessive to call it alternative) logic for solving the problem of free will. Namely, additional opportunities open up when we distinguish between the viewpoints from which this problem is considered. From the *standpoint of a researcher*, the question of free will, as already discussed, is the subject of a hypothesis, and thus the question can remain unanswered for an indefinitely long time. But from the *standpoint of a participant in real social practice*, the problem of the presence/absence of free will needs to be solved almost immediately: making a decision and bearing responsibility for it before the community — performing a deed!

Therefore, in both the academic conceptualization and laypersons’ understanding, the concept of free will is not a magical metaphysical notion, but rather a reference to choice, agency, and unconstrained action (Feldman, 2017).

The transition from the position of the researcher to the position of the (real life) actor necessarily moves us into the sphere of psychology. Note how G. Feldman formulated the definition of the construct under discussion: “The belief in free will is a generalized lay-belief regarding the capacity for human choice — “Do I (and others) have a choice, and if so, can I (and others) freely choose to do otherwise?” As a belief, it captures a mental representation by a person who believes there’s a link between an object, in this case humans, and an attribute, in this case “free will,” or the capacity for choice” (Feldman, 2017, p. 4). Note the grammatical formulation use of the first person (“I”), which reflects the change in position from an observer (third person) to a doer (first person).

But here, too, there is reason to be surprised: Social and psychological studies of free will are conducted as if this freedom of choice is influenced exclusively by social pressure. A typical example of such research is the experimental imposition of alternative beliefs on subjects, testing the presence or absence of free will.

In 2008, K. Vohs and J. Schooler (2008) investigated the relationship between the belief in free will and human behavior. This and subsequent studies by social psychologists have revealed interesting effects, as follows:

*Attenuated free will beliefs* led to:

– Less self-knowledge, such that participants reported feeling more alienated from their true selves and experienced lowered perceptions of authenticity while making moral decisions (Seto, & Hicks, 2016).
– Increased aggression and decreased willingness to help others (Baumeister, Masicampo, & DeWall, 2009).
– Reduced gratitude for help both in the real past and in a hypothetical scenario (MacKenzie, Vohs, & Baumeister, 2014).
– Participants’ being more passive, exhibiting a reduction in intentional engagement (Lynn, Van Dessel, & Brass, 2013).
– Heightened conformity and copying of the opinions of others (Alquist, Ainsworth, & Baumeister, 2013).
– Perceived meaninglessness. Reducing self-awareness to the goal of reducing existential conflict (i.e., conflict related to psychological and philosophical values), thus making conformity the regulatory goal (Moynihan, Igou, & van Tilburg, 2018).

Vice versa, *belief in free will* is linked to feelings of belonging and subjective significance (Moynihan, Igou, & van Tilburg, 2017), the sense of freedom to act, and the conviction that we are the authors of our actions and are actively engaged with the world (Seto, & Hicks, 2016).

There is a strong indication that belief in free will is positively associated with *greater personal maturity* (an empirical verification of this interpretation is presented below). Conversely, belief in determinism when making decisions reduces a *person’s personality potential*.

Perhaps the strongest concomitant of belief in free will is the sense of agency. This is substantiated in detail in the work of G. Feldman, where the key idea is as follows: “The belief in free will is different from other constructs in that it conceptualizes agency as being about the capacity for choice” (Feldman, 2017, p. 5). Here the importance of choice lies in the fact that it is a fundamental factor in understanding the human psyche and a defining feature of human existence.

However, the question remains as to how the construct of “free will” and belief in free will does its constructive work, and what processes it activates to increase a person’s personality potential. In his work, G. Feldman (2017), who views the essence of free will through the concept of choice^2^, turned to the phenomenology of performing actions. This is not by accident, since it is not enough to believe in a construct; you also have to act in accordance with your belief, *i.e.,* make choices. The author paid special attention to a person overcoming constraints in making choices. Actually, it is this overcoming of causal determinants that makes the will free, affirming a person’s subjectivity.

In terms of cultural-historical psychology, the construct “belief in free will” is a cultural tool (psychological tool), “a means of internal activity aimed at mastering oneself ” (Vygotsky, 1978, p. 55), and of a person’s self-creation as a personality which needs self-confirmation, self-realization, and self-actualization. “Free will should be understood not as a philosophical, theological, or biological property of all human action, but rather as a way of operating within culture” (Baumeister, & Monroe, 2014,. 10).

Indeed, in order to be able to exist in his culture, a person needs to have a psychological tool that allows him or herself to show autonomy in relation to hereditary and environmental factors. K. Dabrowski calls free will the “third factor”: “Its activity is autonomous in relation to the first factor (hereditary) and the second (environmental) factor. It consists in a selective attitude with regard to the properties of one’s own character and temperament, as well as to environmental influences” (Dabrowski, 1973, p. 80).

When making a personal choice (deciding to act), biological determinism and social demands are recognized as possible constraints, but are accepted or rejected by means of the construct “belief in free will.” The more active a person’s belief in free will, the more free his will is, both from predetermined biological processes and from the pressure of social prescriptions.

In turn, free will as a social construct is also necessary for society itself to ensure that a person takes personal responsibility for the results of his or her own choices and deeds.

*Research hypotheses:*

1. A free will attitude contributes to an increase in the personal maturity of a person’s actions. A deterministic attitude contributes to a decrease in the maturity of actions.
2. The work of the personality is expressed through overcoming social pressure.

## Methods

Our sample was comprised of 340 people, ages 30–50: 178 (52%) were women and 162 (48%) were men. They were divided into the following groups:

– Group with a deterministic attitude: 113 people, of whom 56 (49.6%) were women, and 57 (50.4%) were men.
– Group with a free will attitude: 112 people, of whom 61 (54.5%) were women and 51 (45.5%) were men.
– Control group: 115 people, of whom 61 (53%) were women and 54 (47%) were men.

### Procedure

Data collection was carried out via the Internet. The respondents’ participation was voluntary and not paid. Before starting work, the subjects received the following message: “Purpose of the study: to find out how people make important decisions for themselves. Please be free in your choices; there are no right or wrong answers. We guarantee that all data will be used only for scientific research purposes in an anonymous form.”

The first group of subjects (having being presented with a deterministic interpretation — no free will) read excerpts from a book by S. Harris (10 quotes). For example: “Our wills are simply not of our own making. Thoughts and intentions emerge from background causes of which we are unaware and over which we exert no conscious control. We do not have the freedom we think we have.”

#### Instructions

“Sam Harris is a PhD in Cognitive Neuroscience and a renowned American publicist and popularizer of science. Below are quotes from his best-selling book *Free Will*, translated into many languages. Read the quotes, comprehend, and evaluate your attitude toward them, where: 1 = definitely disagree; 2 = disagree; 3 = not sure; 4 = agree; 5 = definitely agree.”

The second group (having being presented with a nondeterministic interpretation — free will exists) read excerpts from a book by V. Frankl (10 quotes). For example: “Man is not fully conditioned and determined but rather determines himself by whether he gives in to conditions or stands up to them. Man does not simply exist but always decides what his existence will be, what he will become in the next moment.”

#### Instructions

“Viktor Frankl is an Austrian psychiatrist, psychologist, and neurologist, the creator of logotherapy. Frankl is the author of books that have undergone a fabulous number of reprints in dozens of languages around the world. Below are quotes from his book *Man’s Search for Meaning*. Read the quotes, comprehend, and evaluate your attitude toward them, where: 1 = definitely disagree; 2 = disagree; 3 = not sure; 4 = agree; 5 = definitely agree.”

All the quotes had a vivid emotional and semantic form of expression and unambiguously reflected the author’s position regarding the “free will” construct.

The third control group filled out the P. Oles “Internal Dialogical Activity Scale” adapted by D. Astretsov and D. Leontiev (2015).

In addition, members of all three groups were asked to make a decision in five difficult life situations which required action.

The stimulus material was constructed based on theoretical ideas about the action; five real life situations were formulated, each of which contained a certain choice of confli icting values that required resolution (see details in Dotsenko, Startseva, & Pchelina, 2020). Each situation had six ready-made solutions offered and one open answer, which would involve a personal decision by the subject (which would be subject to expert judgment on the part of researchers about its degree of personal maturity). The level of maturity was assessed in accordance with the types of action decided upon (Dotsenko, 2009; Dotsenko, Startseva, Pchelina, Karaberova, & Ivantsova, 2020) and received a score from 0 to 8, where 0 was the refusal to make a decision and take any action (refusal to perform a deed), and 8 was a creative solution that supported two or more values at once (the most mature act). To translate meaningful choices into figures, standardized coding was used and, in controversial cases, five expert judgments were solicited.

The results were processed using the SPSS Statistics20, STATISTICA10 software.

## Results and Discussion

**Figure 1.**
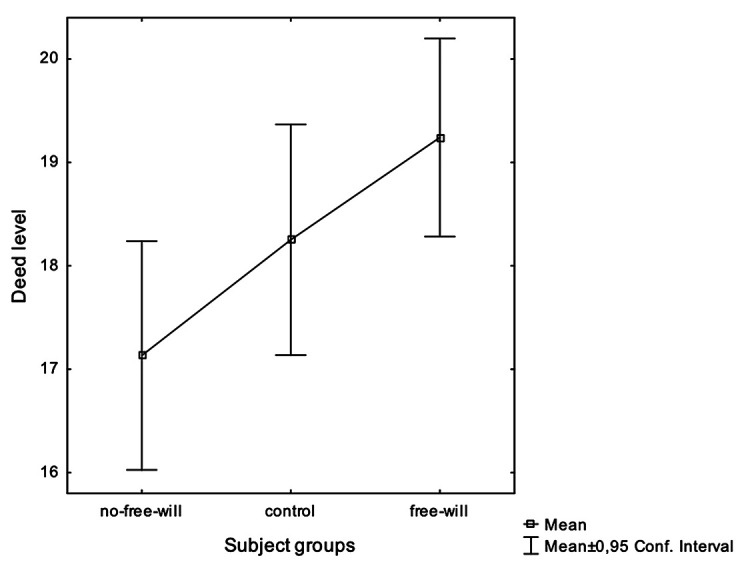
Indicators of the maturity of actions by persons with different beliefs in free will in the three groups of subjects

As we can see in *Figure 1*, the level of maturity of action by the control group is approximately equidistant from the other two. The level of maturity of actions by the subjects who received the set for no-free-will statement is lower than the level of maturity of actions by the subjects who received the set for free will (Mann-Whitney U test 5133; p = 0.014). Therefore, we are entitled to conclude that the statements influenced the formation of the subjects’ attitude, thereby supporting hypothesis 1.

However, the subjects were asked not only to read excerpts from books, but also to express their *attitude* toward the quotations: to accept or reject the messaging. We did not impose a ready-made attitude, but provided an opportunity for the subject to declare (take!) **their position** in relation to the idea of free will. The experimental task served as a model of a real life situation: 1) the subjects were gently pressured in favor of free-will or no-free-will; but 2) they were able to take and express their own positions by scaling their agreement/disagreement with the proposed statements.

Therefore, it is reasonable to pose the following research question: Did all the subjects agree with the statements proposed to them (attitudes towards no-free-will or free will), and what were the levels of actions of those who agreed versus those who protested?

We got the general picture (shown in *Figure 1*), and there were shifts. Now we are not interested in all the subjects, but only those who actively resisted the pressure of “authorities,” and those who (almost) completely agreed with the proposed theses, in other words, our attention is drawn to “tails” of distributions.

**Table 1:** Groups of subjects depending on the attitude to the proposed excerpts from books (sets)

Set Position	free-will	no-free-will
Expressing agreement	“free-will” (+)	“no-free-will” (+)
Expressing disagreement	“free-will” (-)	“no-free-will” (-)

To obtain these fractional groups, we used the filter *Mean* ± *Standard deviation*. Subjects *Min*. ≤ *n* < Mean – *Stv. Dev*. by the parameter of belief in free will formed the “free-will” (–) group, and by the parameter of no-free-will, the group “no-free-will” (–). Subjects *Mean* + *Stv. Dev*. > *n* ≥ *Max*. by corresponding parameters were divided into the groups “free-will” (+), and “no-free-will” (+).

**Figure 2.**
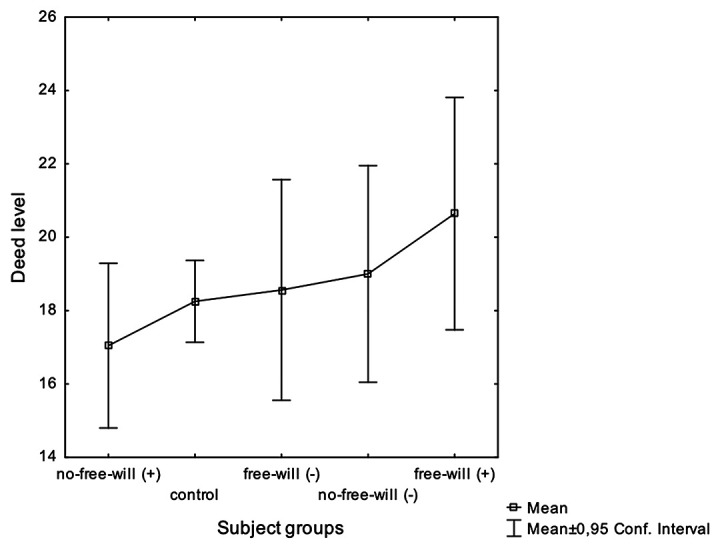
Indicators of the maturity of deeds in persons with different beliefs in free will in the three groups of subjects

The effects of the subjects’ attitudes toward the proposed sets are clearly visible in *Figure 2*. The positive attitudes toward both the idea of no-free-will and the idea of free will, measured by the maturity of their actions, were the most polarized. The level of maturity in the subgroup “free-will” (+) was higher than in the subgroup “no-free-will” (+) at U 90.5, p = 0.041 (the level of significance has become somewhat worse, due to the smaller size of the subgroups).

Disagreement with the experimental sets moved groups of subjects to opposite poles, which is quite meaningful and theoretically expected. So, the “free-will” (-) group is almost equalized with the control, and “no-free-will” (-) is noticeably approaching the free-will pole. This is a very striking result: **the work of the individual overcomes the pressure of the authorities** to whose statements the participants of the experimental groups were exposed. The attitude toward the construct of “free will” is a means by which a person asserts his/her subjectivity. Efforts to overcome the press of circumstances reveal the essence of personal work: the subjects who objected to the set for no-free-will [“no-free-will” (-)], showed a high level of personal maturity of their actions. Consequently, our second hypothesis found its empirical proof.

Thus, belief in free will, its incompleteness (limitedness) or absence, as a person’s value-based position, found its embodiment in the level of personal maturity of his/ her actions. And the more the belief in free will was actualized, the more mature personal choices were made.

## Conclusion

Understanding the results we obtained opens up the possibility of supporting the following ideas.

### Biological reductionism stops us from finding free will

The abundance of efforts to reduce the phenomenon of free will to biological processes — with a wide range of conflicting results — allows us to make two plausible explanations. First, during such reduction, the phenomenon in its essence disappears; biologization kills freedom, which corresponds exactly to the deterministic nature of biological processes. Second, free will cannot be the subject of an ontological approach; it is a phenomenon of an epistemological plan.

### A Social Construct is a psychological tool

Free will as an epistemological reality is a social construct that has the properties of a cultural means (historically conditioned environment). Society uses this means to convince its members to believe in their ability to be free to choose a line of behavior, as well as to encourage a person to take responsibility for the results of their choices.

### The determining action of the “third factor.

Humanity has developed a broad repertoire of means to overcome biological limitations, and go beyond their direct action. One of these means is belief. A person has the ability to accept or reject existing constraints (with an arbitrarily chosen degree of freedom), both biological and social. A mature personality uses the construct of free will as a tool for his/her further personal development and self-actualization (actualization of their essential qualities).

The paradox of free will, revealed in scientific discourse, is solved by each individual person in everyday activities, but with varying degrees of success and personal maturity.

## Limitations

The main limitation of this study was the restriction of the sample’s age to 30-50 years. Therefore, we do not claim applicability of our results to other age groups. Further studies involving people ages 18-30 and older than 50 years can clarify the relationship between the belief in free will and the level of maturity of actions. We will also be able to compare the levels of people’s subjectivity, and their ability to overcome the pressure of authorities in subjects of different age groups.

## Footnotes

1 RP — readiness potential.

2 The term “choice” appears 49 times in the work under discussion.
